# 3 M antimicrobial incise drape combined with MEBO in wound recovery of postoperative hypospadias surgery

**DOI:** 10.1007/s11255-023-03925-2

**Published:** 2024-02-02

**Authors:** Hongyan Li, Junting Li, Xiaoyan Yao, Han Chu

**Affiliations:** 1 Department of Operating Room, Anhui Provincial Children’s Hospital, Hefei, 230000 Anhui China; 2 Department of Urology, Anhui Provincial Children’s Hospital, Hefei, 230000 Anhui China

**Keywords:** Hypospadias, Wound Dressing, MEBO, Child

## Abstract

**Objective:**

To evaluate the efficacy of different dressing methods in wound healing and the postoperative outcome in children who underwent hypospadias repair.

**Methods:**

Altogether 109 children with distal hypospadias who underwent urethroplasty were recruited from our hospital between January 2021 and March 2023. All patients were randomized in two groups according to the different dressing methods: Group A receiving 3 M antimicrobial incise drape + MEBO (moisture-exposed burn ointment) and Group B receiving absorbent dressing + elastic bandage dressing. The age at surgery, operation time, bleeding during the dressing, postoperative changes in glans color, dressing fell off, comfort of children during the dressing, difficulty in dressing removal, and degree of pain during dressing removal were compared between the two groups.

**Results:**

Differences in age at surgery (*p* = 0.337) and operation time (*p* = 0.055) were not significant between the two groups. The overall effectiveness of the dressing was better in Group A than that in Group B. Only five cases in Group A had blood leakage after dressing (*p* = 0.006), and there was no dressing dislocation (*p* < 0.001) or glans color abnormality (*p* < 0.001). Moreover, the number of complication cases was less. The overall comfort and pain degree during dressing removal in Group A was better than that in Group B (*p* < 0.001).

**Conclusion:**

Postoperative dressing using 3 M antimicrobial incise drape + MEBO can achieve lower incidence rates of bleeding during dressing, postoperative glans darkening, and dressing falling off, a lower pain degree during dressing removal, and a better overall comfort level than those of the control group. This method is cost-effective and clinically safe, which contributes to the postoperative recovery of children with hypospadias and is thus worth promoting and applying.

## Introduction

Hypospadias, one of the genitourinary malformations in children, occurs in 1 of 250 live male births [1]. Urethroplasty using a variety of surgical methods is the main treatment for hypospadias. Complications such as urethral fistula, urethral diverticulum, urethral stricture, and incisional infection may take place after hypospadias surgery, and the surgical success rate is affected by various factors [2], such as penile blood abundance, penile intermittent erection, and pediatric strenuous activities. Even if the operation is strictly hemostatic, postoperative bleeding or blood seepage from the penile incision may still occur, followed by the formation of blood scabs. The formed blood scabs will affect the healing and recovery of the incision on the skin and urethral tissues, and are prone to infections, finally resulting in other complications such as urethral fistula and urethral fracture [3]. The expression of postoperative pain and discomfort in the penis of children leading to crying and restlessness will increase the anxiety of the children and their parents, which is not conducive to their recovery [4]. Consequently, appropriate postoperative dressing of hypospadias to minimize blood leakage and scab formation and to increase the comfort and surgical success in children is a clinical problem that needs to be addressed. The present study aimed to compare two different dressing methods to investigate the efficacy of 3 M antimicrobial incise drape + MEBO (moisture-exposed burn ointment) as a dressing method after hypospadias surgery.

## Materials and methods

This prospective single-blinded randomized clinical trial was conducted between January 2021 and March 2023 under appropriate approval by the Institutional Review Board (IRB) and the Ethics Committee of Anhui Provincial Children’s Hospital (approval no. 2019xkj079). The parents of our included children provided the informed consents.

**Fig. 1:**
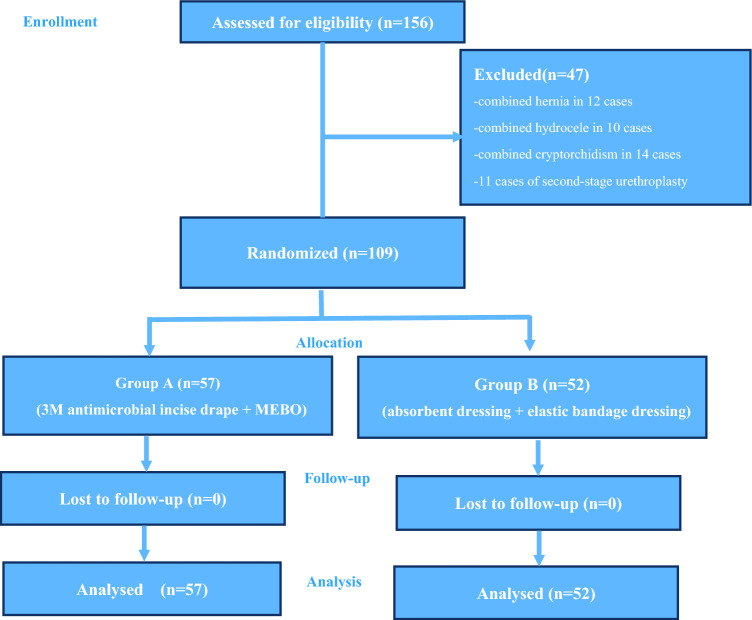
Patient allocation

In total, 109 children with distal hypospadias admitted to the Department of Urology of Anhui Children’s Hospital from January 2021 to March 2023 were included in this study. The children, all of whom were boys, were randomly divided into two groups (Fig. 1). Their average age was 58.56 (range, 7–156) months. All the operations in these children were performed by the same treatment team of surgeons.

Inclusion criteria:

- Children diagnosed with hypospadias with no other underlying diseases.
- Children undergoing one-stage urethroplasty (TIP, Mathieu, Onlay, Inlay, and Duckett).

Exclusion criteria:

- Children with other coexisting diseases, such as cryptorchidism, hydrocele, and DSD (disorders of sex development).
- Those receiving second-stage urethroplasty.

After general anesthesia, the child was disinfected and toweled, and different methods of urethroplasty were performed according to the specifics of hypospadias. There were 60 cases of TIP, 8 cases of Mathieu, 7 cases of Onlay, 13 cases of Inlay, and 21 cases of Duckett. Finally, postoperative penile dressing was wrapped, and the children were randomized to two groups.

**Fig. 2:**
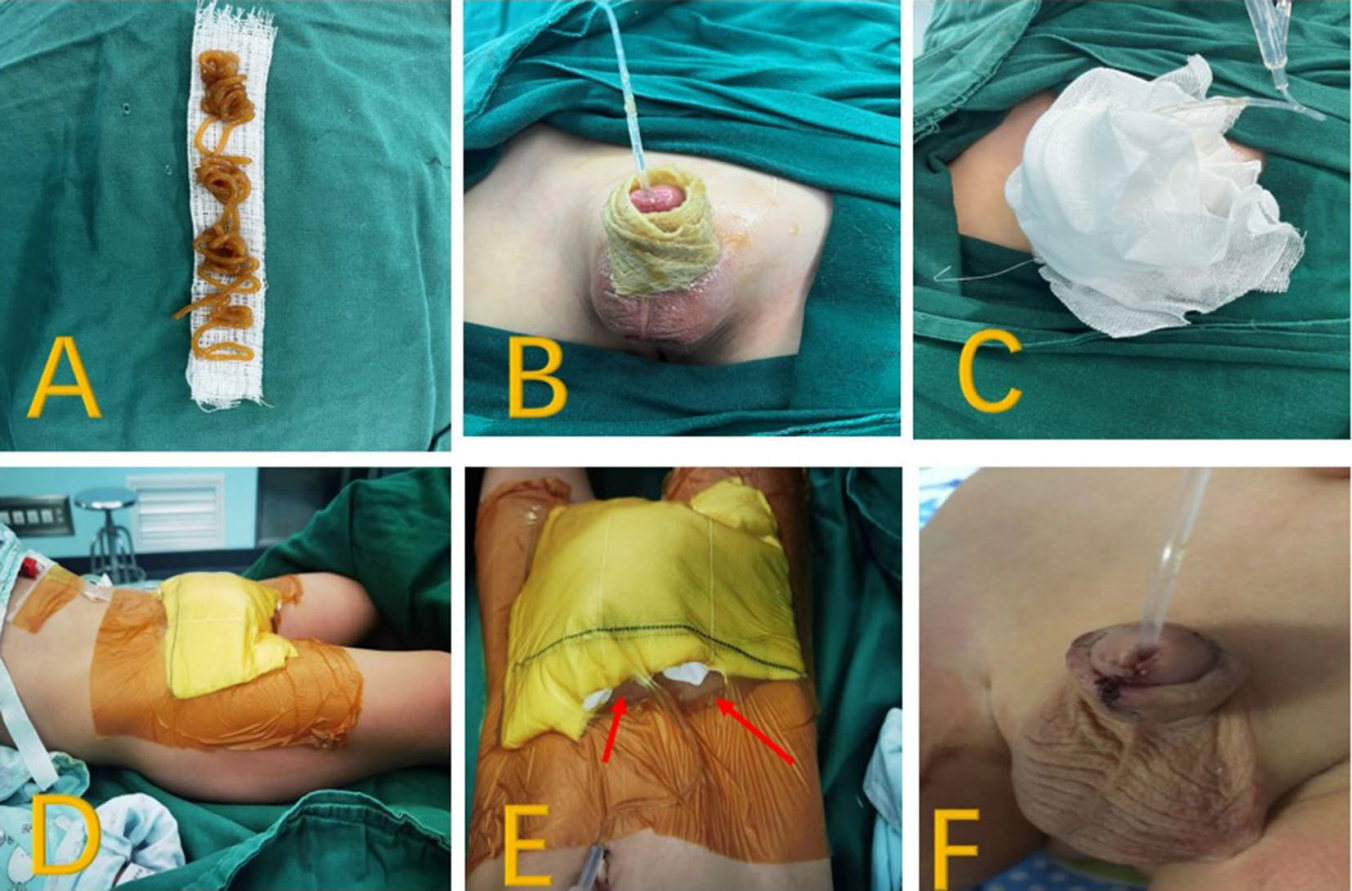
**A** Apply MEBO onto sterile gauze, **B** wrap MEBO-applied sterile gauze around penis, **C** overlaying gauze on the penis, **D** wrap with 3M antimicrobial incise drape, **E** cephalad view, and making two holes for ventilation (red arrow), easy to know the color of the glans. **F** Appearance after dressing removal

In Group A, after the completion of surgery, MEBO was applied on a small sterile gauze and wrapped around the penis, and the penis was gently back stretched so that it was against the prepubic bone. Later, about 4–6 layers of sterile gauze were superimposed on the penis, which was eventually covered with iodine film for pressure fixation. Coverage: below the navel to the upper 1/3rd of the thighs, between the mid-axillary lines on both sides (Fig. 2).

**Fig. 3:**
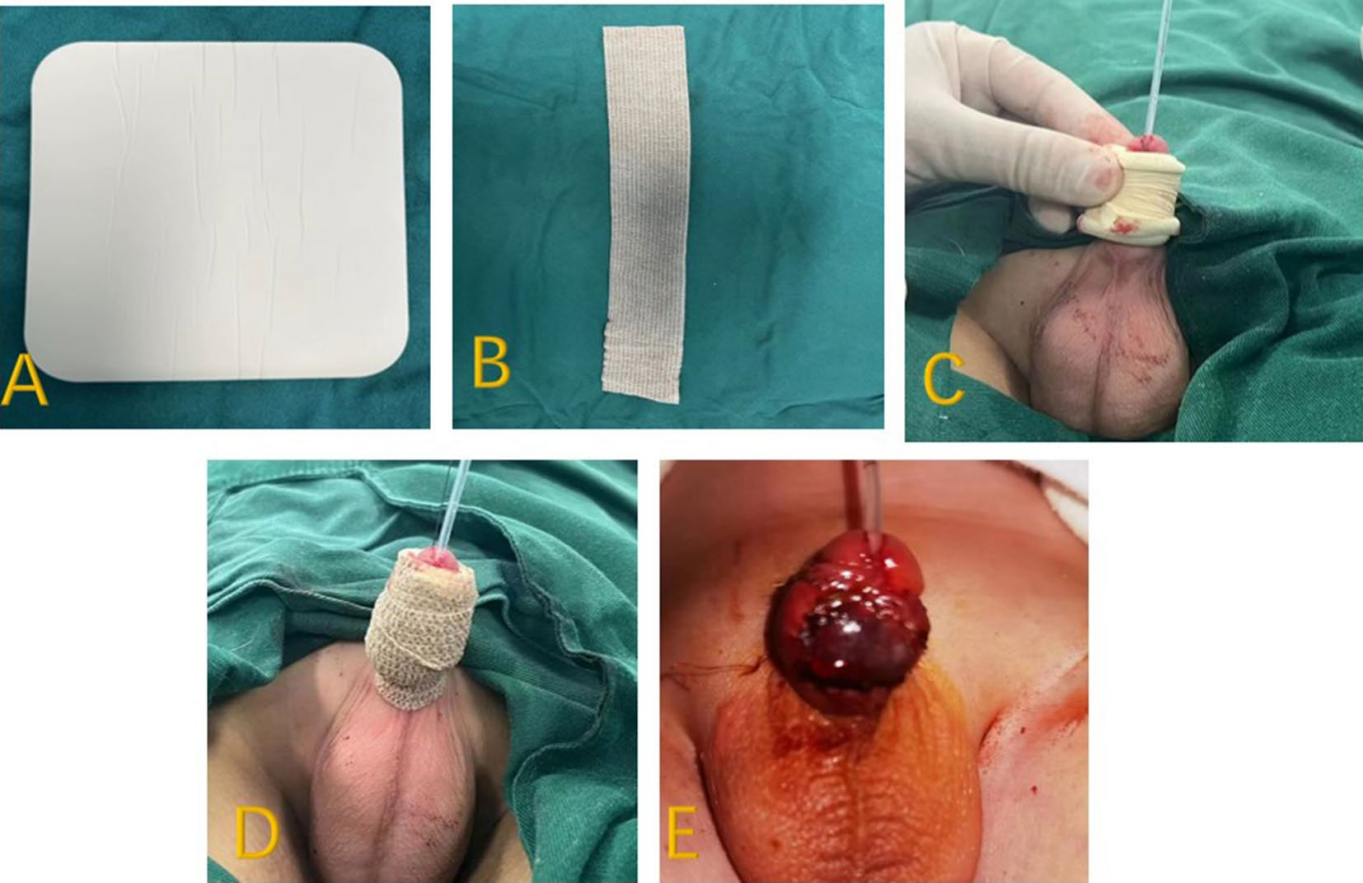
**A** Absorbent dressing, **B** self-adhesive elastic bandage, **C** absorbent dressing of appropriate width wrapped around the penis, **D** self-adhesive elastic bandage to dress the penis, **E** appearance after dressing removal

In Group B, after the operation, the absorbent dressing was cut to the length of the penis and wrapped around it, and then a self-adhesive elastic bandage was wrapped around the penis, which needs some pressure but not overly tight (Fig. 3).

## Postoperative care

Dressing removal was completed in all the children 5 days after surgery. Then, the age at surgery, operation time, the presence or absence of bleeding during the dressing, postoperative changes in glans color, whether the dressing fell off, the comfort of children during the dressing, the difficulty in dressing removal (removal without saline or iodophor disinfectant moistening and soaking was considered to be easy, otherwise it was considered to be difficult), and the degree of pain during dressing removal were compared in children between the two groups.

To be specific, the dressing comfort and dressing removal performance in children were assessed using the FLACC pain scale [5] (also called infant and child behavioral observation). The FLACC pain scale consists of five components, namely, facial expression, leg movement, activity, crying, and consolability, with each component being rated on a scale of 0–2, and the total assessment score ranged from 0 to 10, with a higher score indicating more pronounced discomfort and pain. All the children were followed up for 3 months after surgery to observe the occurrence of postoperative complications, such as the occurrence of urethral fistula, urethral stricture, urethral diverticulum, and incision dehiscence.

## Statistical analysis

Statistical analysis was performed using SPSS 16. Descriptive data were expressed as numbers. The normally distributed continuous variables were analyzed by *t* test, while categorical data were tested by Chi-square test or Fisher’s exact test. *P*-values < 0.05 were considered statistically significant.

## Results

There was no statistically significant difference in age at surgery [(36.33 ± 36.09) vs. (40.10 ± 37.09), *P* = 0.337 vs. *P* > 0.05] or operation time [(121.61 ± 38.26) vs. (108.35 ± 32.57), *P* = 0.055 vs. *P* > 0.05] between the two groups. Compared with Group B, the dressings in all children of Group A were smoothly removed without saline or iodine moistening (*P* < 0.001), with good overall appearance of the penis. The overall comfort and pain degree during dressing removal in children of Group A were better than that of Group B (*P* < 0.001). No darkening of glans (*P* < 0.001) or dressing dislodgment (*P* < 0.001) was observed in Group A. There were five cases of blood leakage after dressing in Group A, but all of them could spontaneously stop and did not need compression or re-dressing. In Group B, there were 16 cases of rebleeding after dressing, which required re-dressing (seven of them due to dislodging of dressing) or compression dressing, and 15 cases of dressing dislodgment after returning to the ward that required re-dressing. Eleven cases of glans darkening were improved by loosening the elastic bandage, and in 17 cases, the dressings had to be removed after moistening with saline or iodine. In Group A, eight cases developed postoperative complications, including two cases (one case was Inlay and the other was Duckett) of postoperative incision infection and dehiscence, five cases of urinary fistula (three in TIP, one in Inlay, and one in Duckett), and one case (Duckett) of urethral stricture. Similarly in Group B, 15 children developed postoperative complications, including nine urinary fistula (eight in TIP, one in Inlay, and one in Duckett), four incision infection and dehiscence (two cases each of inlay and Duckett), and one urethral stricture (Duckett). Moreover, differences in the postoperative complications were not statistically significant between the two groups, but the number of cases was lower in Group A than that in Group B (*P* = 0.065) (see Table 1). In comparison to Group B, we found that penile edema was milder and more homogeneous in Group A while reducing nursing care and parental anxiety. Importantly, the dressing method is also clinically safe since no patients in our series suffered from allergy or intolerance to the product.

**Table 1:** Comparison of results between the two groups

	Group A	Group B	*P*-*value*
**No**	**57**	**52**	
TIP	30	30	
Mathieu	3	5	
Onlay	4	3	
Inlay	7	6	
Duckett	13	8	
**Age**	**36.33 ± 36.09**	**40.10 ± 37.09**	***P* = 0.337**
TIP	30.27 ± 40.16	47.90 ± 43.92	*P* = 0.289
Mathieu	54.33 ± 58.23	26.20 ± 10.50	*P* = 0.309
Onlay	22.00 ± 11.05	33.00 ± 34.07	*P* = 0.562
Inlay	20.29 ± 15.54	40.83 ± 29.65	*P* = 0.137
Duckett	32.23 ± 34.49	21.63 ± 14.09	*P* = 0.421
**Surgery time**	**121.61 ± 38.26**	**108.35 ± 32.57**	***P* = 0.055**
TIP	98.76 ± 14.69	93.07 ± 13.36	*P* = 0.128
Mathieu	85.67 ± 18.88	75,80 ± 16.33	*P* = 0.463
Onlay	206.25 ± 48.18	186.00 ± 18.73	*P* = 0.528
Inlay	137.14 ± 20.32	120.00 ± 14.13	*P* = 0.131
Duckett	148.46 ± 22.67	146.25 ± 22.70	*P* = 0.831
**Bleeding after dressing**	**5**	**16**	***P* = 0.006**
Color of glans after dressing*	**0**	**11**	***P* < 0.001**
**Dislodgment of dressing**	**0**	**15**	***P* < 0.001**
**Postoperative complications**	**8**	**15**	**P = 0.065**
TIP	3	8	
Mathieu	0	0	
Onlay	0	0	
Inlay	2	3	
Duckett	3	4	
**Comfort during dressing (FLACC)**	**0.60 ± 0.49**	**2.50 ± 1.31**	***P* < 0.001**
TIP	0.43 ± 0.50	2.13 ± 1.07	*P* < 0.001
Mathieu	0.67 ± 0.58	2.20 ± 0.45	*P* = 0.005
Onlay	0.75 ± 0.50	5.33 ± 1.15	*P* = 0.001
Inlay	0.71 ± 0.49	2.17 ± 0.75	*P* = 0.002
Duckett	0.85 ± 0.38	3.63 ± 0.52	*P* < 0.001
**Cases of difficulty in removing dressings**^#^	0	17	***P* < 0.001**
**Performance of the child at dressing removal (FLACC)**	**4.84 ± 0.84**	**8.29 ± 1.00**	***P* < 0.001**
TIP	4.73 ± 0.91	8.83 ± 0.46	*P* < 0.001
Mathieu	5.00 ± 1.00	7.40 ± 0.58	P = 0.004
Onlay	4.50 ± 0.58	8.00 ± 1.00	*P* = 0.002
Inlay	4.86 ± 0.69	6.67 ± 1.37	*P* = 0.010
Duckett	5.15 ± 0.80	8.13 ± 0.64	*P* < 0.001

Bold indicates the results of the main items to be compared, which appears to be eye-catching. In contrast, non-bold is the comparison of subgroups of the main items

*A dark red glans is considered abnormal

^#^Removal after infiltration with saline or iodophor is considered difficult and removal without is easy

## Discussion

An ideal dressing method needs to have a certain absorption capacity, appropriate compressive hemostatic effect without affecting the blood supply of the tissues, as well as being easy to remove but not easy to dislodge on their own, improving the child’s postoperative comfort and reducing the pain of removal. Many reports have been published on postoperative dressing of hypospadias, and there is no uniform standard.

Various dressing methods are available such as a bacterial cellulose dressing, a multiperforated pellicle dressing made by polysaccharide, installing vacuum sealing drainage (VSD) after the use of Mepilex bandage, applying antiseptic cream of framycetin + gauze pieces + dressing with Dynaplast circumferentially all over the penis, using tubular finger oxygen-enriched oil inside the coated device, and using compound chamomile and lidocaine hydrochloride gel or autologous platelet gel [6–11]. Each has its own advantages and disadvantages. However, not all dressings are available in all countries, so finding the right one for your circumstance is the best option.

We derived the bandaging for Group B by drawing on the bandaging reported by *Singh *et al. [12]. In this method, involuntary erection of the penis can lead to elongation and retraction, which may induce retraction of the penile skin and lead to uneven skin force, thereby causing possibility of partial flap ischemia and necrosis. Similarly, if the dressing is excessively tight, it will lead to poor blood supply of the flap, which is thus susceptible to urinary fistula and incisional dehiscence. Postoperative dressing dislodgment may cause postoperative bleeding, and localized hematoma or formation of blood scabs can easily induce infection under the scabs, leading to incision cracking or urinary fistula [13].

MEBO (moisture-exposed burn ointment) is a Chinese burn ointment with a USA-patented formulation since 1995, which was developed at the China National Science and Technology Centre in Beijing in 1989 [14]. It possesses diverse pharmacological activities, such as clearing heat and removing toxins; promoting tissue regeneration; preventing adhesion, anti-inflammation, analgesia; and promoting wound healing. As a result, it has been extensively used in various disciplines, and its safety has been confirmed by many parties [15].

We applied 3 M antimicrobial incise drape + MEBO that can reduce postoperative pain (incisional pain or distraction pain to varying degrees) and discomfort in children and prevent detachment of the urinary catheter, which reduces the worries of the parents about their children. Meanwhile, it also reduces the clinical nursing burden. It is possible that the glans may not be visualized when the child has a small penis, but do not be concerned, as this type of dressing provides an even pressure on the penis and does not cause excessive pressure to cause glans ischemia, which was not seen in our study. 3 M antimicrobial incise drape + MEBO creates a moist physiological repair and growth environment for the wound after hypospadias surgery, keeps the wound in a moist state, and reduces the pain and discomfort caused by penile incision, which is also in line with the concept of wet healing proposed by Hinman [16]. This kind of moist healing environment between the wound and the dressing is mionectic, is slightly acidic, is close to the body temperature, and maintains the wound in a constant moist state that is close to the physiological state. This is conducive to the growth of the granulation tissue and the division and proliferation of cells, and may reduce the risk of blood scab formation, which promotes complete wound healing and shortens the time of wound healing [17]. Therefore, do not be concerned when it appears that part of the dressing is soaked with urine, as this does not affect the child’s comfort or the healing of the incision.

From our clinical observation, the following advantages are discovered from the application of 3 M antimicrobial incise drape + MEBO in the postoperative dressing of congenital hypospadias: a) This dressing method can achieve a good anti-adhesion effect, reduce the difficulty in dressing removal, and play a role in reducing postoperative pain, with good comfort. b) It maintains moist wound environment and contains certain herbal ingredients to promote wound healing. c) Relative isolation from the environment reduces the risk of infection and prevents the penis from being touched by accident, thereby providing the “helmet-like” protection. D) Reduces dilemma of whether the penis is in an erect state or not, and no dressing will fall off and does not cause rebleeding. e) The overall force on the penis is uniform, which does not easily impair the blood supply of the skin flap, and the overall appearance is satisfactory due to the mild postoperative glans and penis edema. f) It is inexpensive (costs about 30 CNY) and easy to apply.

## Conclusion

In conclusion, postoperative dressing using 3 M antimicrobial incise drape + MEBO is inexpensive, highly safe, and effective and the postoperative appearance is more beautiful, which also can reduce postoperative penile edema and promote wound healing in children undergoing hypospadias repair surgery. This method of dressing provides good comfort and is easy to remove. To a certain extent, the occurrence of postoperative complications can be reduced. This method of dressing is worth promoting for its application.

### Limitation

There are some limitations in this study; for instance, the sample size of various surgical methods was small and the follow-up period was short. In future, we will further explore its real efficacy using other surgical methods.

## Data Availability

The datasets used and analyzed during the current study are available from the corresponding author on reasonable request.
